# Flexible and expeditious assay for quantitative monitoring of alpha-amylase and amyloglucosidase activities

**DOI:** 10.1016/j.mex.2019.01.007

**Published:** 2019-01-24

**Authors:** Hugo M. Oliveira, Alice Q. Pinheiro, António J.M. Fonseca, Ana R.J. Cabrita, Margarida R.G. Maia

**Affiliations:** REQUIMTE, LAQV, ICBAS, Instituto de Ciências Biomédicas de Abel Salazar, Universidade do Porto, Rua Jorge Viterbo Ferreira, 228, 4050-313, Porto, Portugal

**Keywords:** Determination of the alpha-amylase and amyloglucosidase activities, Enzyme activity, Starch-iodine, Glucose oxidase/peroxidase, Glucose yield

## Abstract

The monitoring of the activity of alpha-amylase and amyloglucosidase is an important tool for studying their role in the hydrolysis of starch. Here we introduced an improved method capable to measure the activity of alpha-amylase and amyloglucosidase from different sources based on a quantitative starch-iodine assay. The developments of the assay sought the consistent preparation of the reagents, the rescale of the assay and the adjustment of the sensitivity. This was complemented by a glucose yield assay for amyloglucosidase that allowed a secondary source of information when insoluble starches were studied. The proposed method showed high precision in long-term use (RSD < 6.3%). Furthermore, all experimental conditions can be adapted according to the equipment available at each laboratory, transforming this method in a broadband analytical tool for screening alpha-amylase and amyloglucosidase activities.

• Tailorable assay based on the starch-iodine staining for the determination of alpha-amylase and amyloglucosidase activities.
• Enhanced consistence of reagent preparation.
• High intra-day and inter-day reproducibility.

## Graphical abstract

**Figure:**
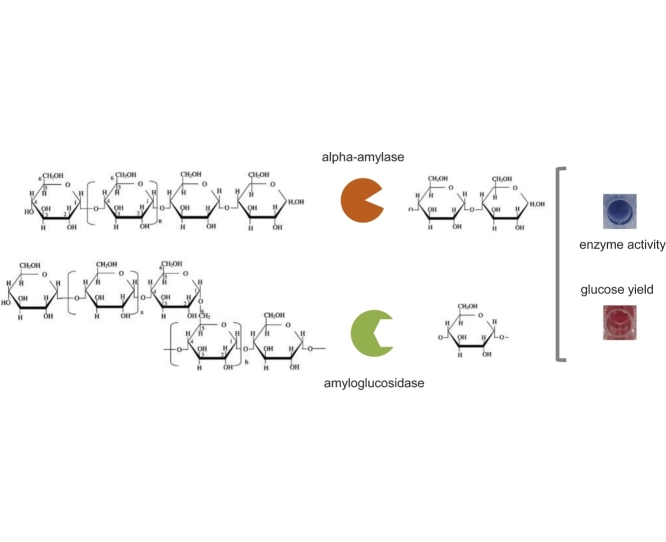

**Specifications Table**

**Table:** 

**Subject Area**	Chemistry
**More specific subject area:**	Analytical Biochemistry
**Method name:**	Determination of the alpha-amylase and amyloglucosidase activities
**Name and reference of original method**	A quantitative starch-iodine method for measuring alpha-amylase and glucoamylase activities. Xiao et al. [5]. Anal. Biochem., 351 (2006) 146–148.
**Resource availability**	N/A

## Method details

### Background

Starch is a widespread raw material with several application fields that cover food (including confectionery and drinks), feed, pharma, chemical, and paper industries [1]. In most of these applications, starch is submitted to a hydrolysis process that aims the conversion of the polymeric chain composed by two high-molecular weight units (amylose and amylopectin) into its primary units (glucose) [2] mediated by amylolytic enzymes. Among the amylolytic enzymes, which act on starch’s polymeric chain as well in its related oligo- and polysaccharides, alpha-amylase, and amyloglucosidase are the most widespread biocatalysts used in the hydrolytic process [2]. It is then important to control the activity of these enzymes in two major complementary roles: as a quality control tool, for monitoring enzyme activity along time and batch to batch reproducibility in different industrial and laboratory applications, and also as a research tool, for studying novel strategies to improve the efficiency of the overall process.

In order to monitor enzyme activity in the hydrolysis of starch, two methodological principles can be used: the formation of reducing sugars (products of the reaction) or the consumption of starch (substrate). The first approach relies on the measurement of reducing sugars, being the dinitrosalicylic acid (DNS) method [3] the classic approach. The DNS method comprises a complex and labor-intensive protocol that includes heating and the use of potentially harmful reagents (*e.g.* phenol). On the other hand, the measurement of the substrate’s consumption is possible using the starch-iodine staining. This method is based on the binding of iodine to terminals of the starch’s polymeric chain [4] that results in a blue colored complex that can be also quantitatively monitored by UV–vis spectrophotometry [5]. In contrast to the DNS method, the staining is instantaneous with the simple addition of a staining solution containing I_3_^−^ that results from the stepwise dissolution of KI and I_2_ in water.

This methodological approach can be used for measuring the activity of both alpha-amylase and amyloglucosidase [5]. The analytical protocol can also be conducted in mild conditions (room temperature), bypassing the heating steps and the protocol complexity that is present in the DNS method, as well in other alternative methods for the same analytes [6].

In this context, we herein describe an adaptation of a method proposed by Xiao et al. [5] for measuring the activity of alpha-amylase and amyloglucosidase using the starch-iodine assay principles. Our developments initially aimed the monitoring of the effect of ultrasound (US) in the activity of these two enzymes [7,8], and sought the improvement / addition of methodological features that lead to complementary information (glucose yield) about amyloglucosidase using a similar experimental protocol. Therefore, the objectives of this work were: (i) the consistent preparation of some reagents, especially the soluble starch and KI / I_2_ solution that generates I_3_^−^, to improve batch to batch reproducibility and maximize the precision and accuracy of the of the starch-iodine method; (ii) the rescale of the activity assay, in order to minimize evaporation issues typical from microplate assays and to adapt it to different experimental requirements (in our particular case this was related with the US-assisted hydrolysis of starch); (iii) the adjustment of sensitivity by modifying the volumes of the starch-iodine microplate assay, to avoid precipitation and to minimize potential deviations to the Lambert’s-Beer law of the absorbance measurements; and (iv) the measurement of glucose yield for amyloglucosidase through the glucose oxidase / peroxidase (GOPOD) method replicating the sample handling protocol used for measuring its activity, but using pure starches from different botanical sources instead of soluble starch.

### Reagents

Soluble starch (PN: S9765), starch from corn (PN: S4126), starch from potato (PN: S4251), starch from rice (PN: S7260), starch from wheat (PN: S5127, unmodified), I_2_ (PN: 207772, ≥ 99.8%), 85% (w/w) *o*-phosphoric acid (PN: 79620), and acetic acid (PN: 695092, ≥ 99.7%) were purchased from Sigma-Aldrich (St. Louis, MO, USA).

D-Glucose (PN: 8337) and KI (PN: 105043) were purchased from Merck (Darmstadt, Germany). Boric acid (PN: A79-212, ≥99.5%) and NaOH (PN: S/4920/60) were purchased from Thermo-Fisher Scientific (Waltham, MA, USA). 37% (w/w) HCl (PN: 131020) was purchased from Panreac (Barcelona, Spain). Glucose oxidase / peroxidase (GOPOD) (K-GLUC) assay kit was purchased from Megazyme (Wicklow, Ireland).

Some examples of amylolytic enzymes assessed by this method were: amyloglucosidase from *Aspergillus niger* (PN: A9913) (illustrative results with this enzyme will be shown in the next sections), and alpha-amylase from *Bacillus licheniformis* (PN: A3306, heat-stable), purchased from Sigma-Aldrich, amyloglucosidase Spirizyme® Achieve and alpha-amylase from *B. licheniformis* Liquozyme® SC DS (AA-2), supplied by Novozymes (Bagsvaerd, Denmark). Detailed information is available in our previous publications [7,8].

### Solutions

All solutions were prepared with ultra-pure water (maximum conductivity of 0.055 μS cm^−1^).

Soluble starch stock solution 2.00 g L^−1^: disperse 1.000 g of soluble starch in ˜5 mL of cold water, add to a beaker with ˜400 mL of boiling water under continuous stirring, after ˜5 min switch off the heat and keep the stir until the solution reach room temperature, transfer the solution to a 500 mL volumetric flask, and complete the volume with water up to 500 mL; keep the solution at 4 °C up to three days. Regarding the potential for structural modification of starch that may affect enzyme hydrolysis and microbial contamination during storage, the users should perform control experiments to check potential problems. If necessary, the solution should be prepared fresh for daily use. *Note: a clear solution shall be obtained.*

Soluble starch standard solutions: prepare by stepwise dilution of the starch stock solution with water; prepare fresh daily.

KI/I_2_ solution: KI 2.0 g L^−1^, I_2_ 200 mg L^−1^. Dissolve 200.0 mg of KI in ˜60 mL of water, after complete dissolution, add 20.0 mg of I_2_ and complete with water up to a total volume of 100 mL; keep at room temperature protected from light. *Note: sonication by an ultrasound bath accelerate the complete dissolution of I_2_.*

Universal buffer: *o*-phosphoric acid 47 mmol L^−1^, acetic acid 50 mmol L^−1^, boric acid 50 mmol L^−1^. Add 2.91 mL of 85% (w/w) *o*-phosphoric acid, 2.86 mL of acetic acid, and dissolve 3.09 g of boric acid in water and complete to a final volume of 1000 mL; keep at 4 °C. *Note:* pH *value must be adjusted to the desired value with NaOH 4.0 mol* *L^−1^ for each particular assay at room temperature.*

NaOH 4.0 mol L^−1^: dissolve 16.0 g of NaOH in water to a final volume of 100 mL; keep at room temperature in a plastic bottle / container.

D-Glucose stock solution 1.000 g L^−1^: dissolve 100.0 mg of D-glucose in water to a final volume of 100 mL; keep the solution at 4 °C up to three days.

D-Glucose working standard solution 200.0 mg L^−1^: dissolve 10.0 mL of D-glucose stock solution in water to a final volume of 50.0 mL; prepare fresh daily.

Amylolytic enzyme working solutions: prepare by stepwise dilution of the commercial solutions with universal buffer at the desired pH to fit the linear range of soluble starch determination. Some examples are reported in our previous publications [7,8]. *Note: amylolytic enzyme working solutions should be prepared fresh daily and kept on ice at all times.*

HCl 1.0 mol L^−1^: add 8.8 mL of HCl 37% (w/w) solution to ˜80 mL of water, mix well, let cool down to room temperature and add water to a final volume of 100 mL; keep at room temperature.

Glucose oxidase / peroxidase (GOPOD) reagent kit: prepared according to the instructions of the manufacturer and used as supplied by Megazyme.

### Materials

Amber glass vials (15 mm × 45 mm × 8 mm, *e.g.* PN: 27217, Sigma-Aldrich).

96-well flat-bottom microplates (well volume ˜340 μL, Thermo Fisher Scientific).

### Instrumentation/apparatus

Magnetic stirrer/heater plate (MAG-H, Gerhardt, Königswinter, Germany), for preparing the soluble starch solution.

Block heater (Stuart SBH130D/3, Staffordshire, UK), for conducting the activity assay with the 4 mL vials. *Note: other heating sources (e.g. water baths) and containers (e.g. eppendorfs) can be adapted according to the equipment available at the laboratory.*

Centrifuge (Astor 8, Astori Tecnica, Poncarale, Italy), for separating the solid particles in the glucose yield assay. *Note: this is a non-interchangeable rotor centrifuge (350* g) *where the vials used for the activity assay can be directly centrifuged (stacked in pairs). Other equipment can be used with adjustments of the centrifugation time and force. The transfer of the vial content to another tube / container may also be necessary.*

Synergy HT reader (Bio-Tek Instruments, Winooski, VT, USA) controlled by Gen 5 software (Bio-Tek Instruments), for measuring the absorbance of the wet-chemistry assays under microplate format. For the starch-iodine method, absorbance measurements were conducted at 580 nm, whereas for the GOPOD assay, the wavelength was set at 505 nm. *Note: if necessary (e.g. for simple microplate readers with optical bandpass filters), the detection wavelength can be adjusted to other values nearby the maximum absorption region of the colored compound (see* Fig. 2, Fig. 4 *for potential alternative wavelengths). In that case, the sensitivity of the determination will be modified.*

### Protocol

#### Amylolytic enzyme activity assay (Fig. 1)

1 Transfer 1500 μL of 2.000 g L^−1^ soluble starch stock solution and 1500 μL of universal buffer (control) or of amylolytic enzyme solution (assay) into a 4 mL amber glass vial. *Note: The amylolytic enzyme solution has to be diluted in order to fit the linear range of the assay. Illustrative dilution factors used with commercially available enzymes are detailed in our previous publications* [7,8]*. It is also possible to use enzymes extracted from other sources, such as flours or microbial preparations* [9]*. More details can be found at* Appendix A.
2 Place the vial in the block heater (or other alternative heating source) previously set at the target temperature of the study during the desired time of the assay (*e.g.* 10 min). *Note: the correcting timing of the assay is critical to reaching high precision. For this reason, the assay time should be longer enough to accommodate minor differences in the timing of the addition of the different solutions. We adopted a time of 10 min in our previous experiments* [7,8]*. Performing each assay in duplicate is also advisable.*
3 Immediately stop the hydrolysis process by adding 750 μL of 1.0 mol L^−1^ HCl.
4 Prepare the soluble starch standards as described in Table 1. *Note: this step can be placed at another point of the workflow if wished.*

**Table 1:** Preparation of soluble starch standards for quantification of amylolytic enzyme activity.

Soluble starch mass/mg	Starch stock solution 2.00 g L^−1^/μL	H_2_O/μL	HCl 1 mol L^−1^/μL
0.00	0	3000	750
0.75	375	2625	750
1.50	750	2250	750
2.25	1125	1875	750
3.00	1500	1500	750
5 Transfer 50 μL of the mixture (sample or standard), 150 μL of water, and 50 μL KI/I_2_ solution to a well of the microplate. *Note: readout replicates should be performed in triplicate. Each standard and sample should be transferred to three wells.*
6 Place the microplate in the plate reader, shake the plate (for ˜30 s using the shaker function of the microplate reader) and read the absorbance at 580 nm, at room temperature. The linear range of the measurements included masses of soluble starch up to 3.00 mg (see Fig. 2 for more details).

**Fig. 1:**
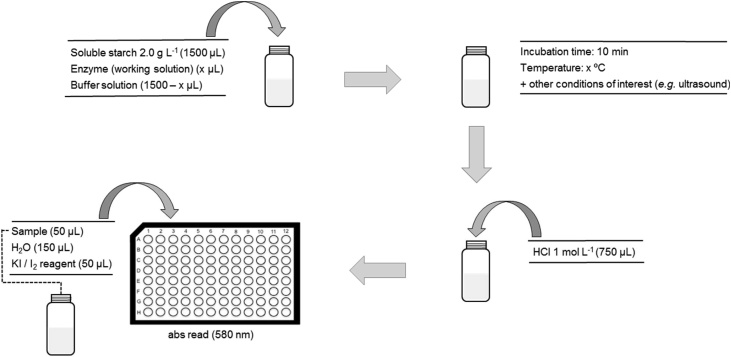
Schematic illustration of the workflow proposed for the quantification of the activity of alpha-amylase and amyloglucosidase. All experimental details are described in the section “Amylolytic enzyme activity assay”.

Adapted from [8].

**Fig. 2:**
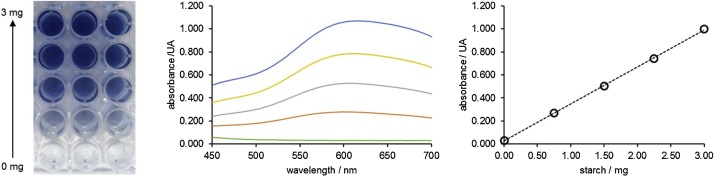
The final aspect of the microplate, absorbance spectra, and illustrative calibration curve of different soluble starch standards (from left to right, respectively) of the starch-iodine quantitative assay used for the characterization of alpha-amylase and amyloglucosidase activities. The masses of soluble starch represented are 0.0, 0.75, 1.50, 2.25, and 3.00 mg.
7 The amount of hydrolyzed starch was the difference between the masses calculated from the calibration curve obtained by plotting absorbance *vs.* mass of starch (Fig. 2) for the control (starch without enzyme, m_control_) and assay (starch with enzyme, m_assay_). For the applied experimental conditions, enzyme activity corresponded to the starch hydrolysis rate expressed in mg of soluble starch per minute (Eq. (1)), where time corresponds to the hydrolysis time in minutes, volume corresponds to the volume of enzyme used for the assay in mL (for a maximum of 1500 μL), and dilution factor corresponds to the total dilution applied to the commercial solution of enzyme. A guided example for calculating the enzyme activity can be found in the Appendix B section.

(1) $$activity\;\left(\text{U}\;\text{m}{\text{L}}^{-1}\right)=\;\frac{m_{control}\;-\;m_{assay}}{time\;x\;volume}\;x\;dilution\;factor$$

#### Glucose yield assay (Fig. 3)

1 Weight 3.00 mg of pure starch into 4 mL amber vials.
2 Transfer 1500 μL of water and 1500 μL of universal buffer (control) or of amyloglucosidase solution (assay) into a 4 mL amber glass vial. *Note: the concentration of the amyloglucosidase solution should be established according to the linearity of the assay. If the objective is to obtain complementary information about the activity, the same concentration used to determine activity can be used* [8].
3 Place the vial in the block heater (or other alternative heating sources) previously set at the target temperature of the study during the desired time (*e.g.* 10 min). *Note: based on the same principles of the enzyme activity assay, the correct timing of the assay is critical to reaching high precision. For this reason, the assay time should be longer enough to accommodate minor differences in the timing of the addition of the different solutions. We adopted a time of 10 min in our previous experiments* [7,8]*. Performing each assay in duplicate is also advisable.*
4 Immediately stop the hydrolysis by adding 200 μL of 1.0 mol L^−1^ HCl.
5 Centrifuge the vials for 5 min at 350 g, at room temperature. *Note: centrifugation settings should be defined according to the equipment available. The objective of this separation is only to remove suspended particles that may interfere in the optical measurements.*
6 Prepare D-glucose standards as described in Table 2. *Note: this step can be placed at another point of the workflow if wished.*

**Table 2:** Standards for quantification of glucose yield.

Glucose mass/μg	D-Glucose working solution 200 mg L^−1^/μL	H_2_O/μL
0.00	0	50
2.00	10	40
4.00	20	30
6.00	30	20
8.00	40	10
10.0	50	0
7 Transfer 50 μL of sample supernatant (or standard), and add 150 μL of GOPOD reagent to each well of the microplate. *Note: readout replicates should be performed in triplicate. Each standard and sample should be transferred to three wells. In some cases, dilution of the sample can be necessary in order to fit the linear range of the calibration curve.*
8 Place the microplate in the plate reader, shake the plate (for ˜30 to 60 s using the shaker function of the microplate reader), incubate for 30 min at 37 °C, and read the absorbance at 505 nm. See Fig. 4 for more details. *Note: if the microplate reader temperature control is unavailable and / or to confirm that the reaction is complete, absorbance can be monitored along the time. At the reaction’s end-point absorbance values should be constant.*

**Fig. 3:**
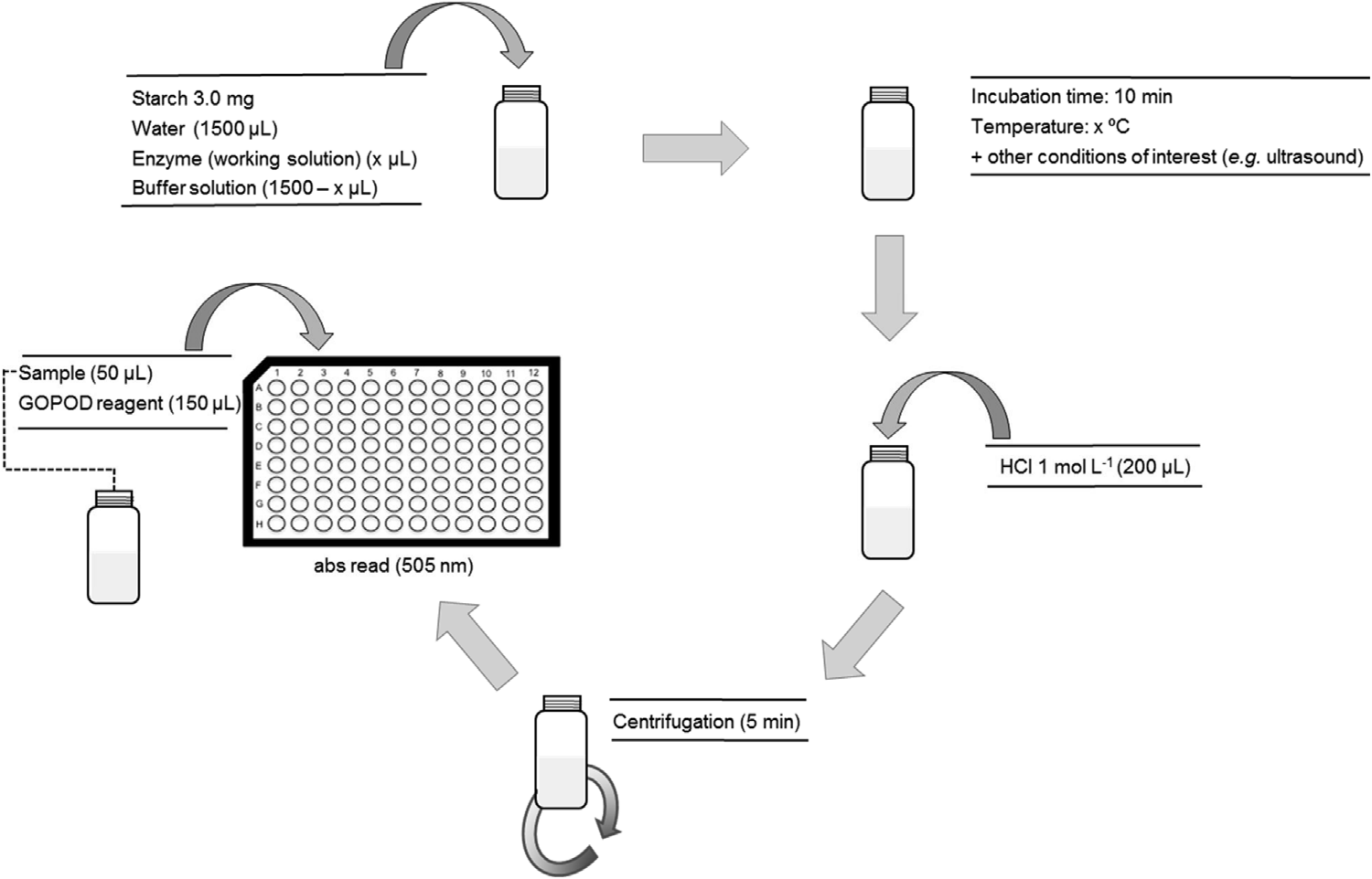
Schematic illustration of the workflow proposed for the determination of the glucose yield when pure starches were submitted to the action of amyloglucosidase. All experimental details are described in the section “Glucose yield assay”.

Adapted from [8].

**Fig. 4:**
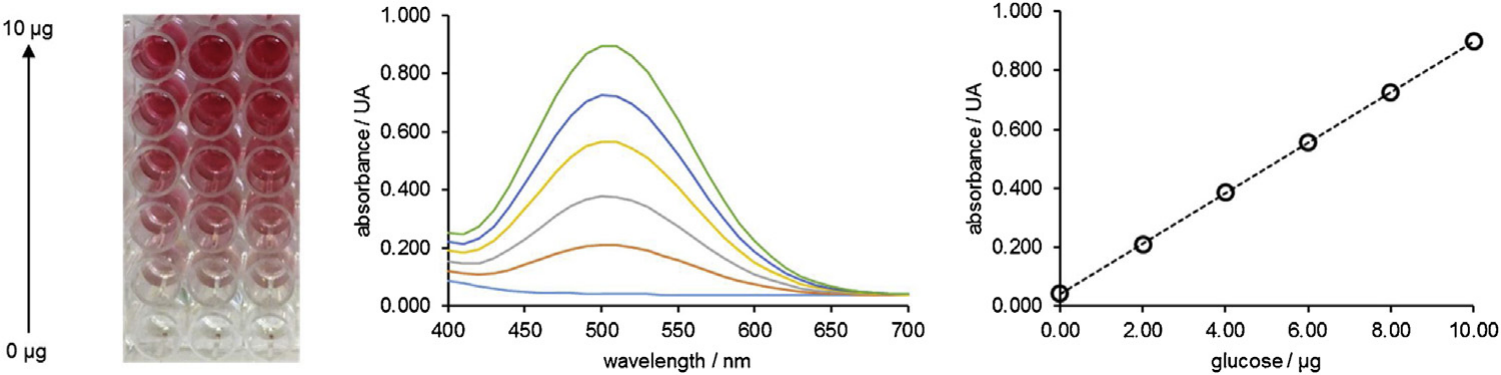
The final aspect of the microplate, absorbance spectra, and illustrative calibration curve of different glucose standards (from left to right, respectively) of the glucose oxidase/peroxidase assay used for the determination of the glucose yield after hydrolysis of pure starches catalyzed by amyloglucosidase. The masses of glucose represented are 0.00, 2.00, 4.00, 6.00, 8.00 and 10.0 μg.
9 The calibration curve for glucose was linear for masses of glucose up to 10 μg (equivalent to concentrations up to 200 mg L^−1^). Glucose yield was expressed as the ratio (expressed in percentage) between the mass of glucose measured after the amyloglucosidase-based hydrolysis process (m_glucose_), and the initial mass of starch (m_starch_), using the same mass units (Eq. (2)). An illustrative example for the calculation is available at the Appendix B section.

(2) $$glucose\;yield\;\left(\%\;weight\right)=\;\frac{m_{glucose}}{m_{starch}}\;x\;100$$

### Additional information

#### Method development

The proposed method relies on principles that allow a fast, simple, and versatile measurement of the activity of alpha-amylase or amyloglucosidase. The development work herein reported sought to answer to the major methodological difficulties that we experienced for implementing the protocols for measuring enzyme activity (for both amylase and amyloglucosidase) and glucose yield (for amyloglucosidase), starting from the report of Xiao et al. [5].

Regarding reagents, we describe in detail the preparation of the solutions of soluble starch and triiodide (from the KI / I_2_ solution), which are key elements for the starch-iodine method. Hence, a reliable protocol for preparing the starch solution is an important aspect regarding the need for a batch to batch reproducibility. The preliminary dissolution in cold water, followed by the dissolution in hot water, and then the mixing and cool down (maintaining the stirring) are critical aspects to obtain a clear (and not cloudy) solution of soluble starch. The use of soluble starch also allowed the standardization of the working conditions, avoiding any influence of the solubility of the starch in the final result. On the other hand, the triiodide solution implies the first dissolution of KI followed by I_2_. We used a US bath to facilitate the complete solubilization of I_2_ and subsequent formation of triiodide (please note that the concentration of KI described in the original report of Xiao et al. is incorrect [10]).

The use of a suitable volumetric scale for the assay was also an important concern for our development work. Hence, we kept the same volume ratio of the original method but we increased the assay to a total volume of 3.00 mL according to our requirements, which were defined by the specifications of the US probe used to study the effect of US in the activity of amylolytic enzymes [7,8]. This also means that the volumes could be easily adjusted in order to match the requirements of each particular enzyme analysis or study. Although the small volume (100 μL) used in the original formulation of the assay [5] is an interesting option to minimize reagent consumption and waste disposal, the high temperatures that these enzymes can be submitted may pose problems of volume control caused by evaporation, which may have negative impact in the precision and accuracy of the method. Furthermore, by performing the assay on a volume scale compatible with eppendorfs or vials, it is also possible to easily transfer the sample other subsequent experimental steps (*e.g.* centrifugation). We adopted this strategy to separate the solid particles that remained after the hydrolysis of different pure starches mediated by amyloglucosidase (glucose yield protocol, step 5).

Concerning to the measurement of soluble starch, the adjustments covered two complementary aspects: bypassing the precipitation found in microplate wells when the original volumes were used, and minimizing potential deviations of Beer’s law when high values of absorbance (above 1) described in the original method were found. The solution for bypassing the precipitation of the starch-iodine complex was diluting the starch standard/sample with 150 μL of ultra-pure water followed by the addition of 50 μL of KI / I_2_ solution (triiodide) reagent (amylolytic enzyme activity assay, step 7). When compared with the original method, these new conditions lead to an extra dilution of the sample (a dilution factor of 5 compared to a dilution factor of 2) and to a longer optical path due to the higher volume placed at each well of the microplate (250 μL *vs.* 150 μL, and assuming the use of a classic cylindrical-shaped flat bottom microplate). Therefore, we were capable to operate in maximum absorbance values close to 1, avoiding potential deviations of Lambert’s Beer law with impact in the analytical performance of the method [11], and simultaneously keeping a linear range compatible with the starch levels found after enzyme-assisted hydrolysis. It is also important to note that the absorption properties of the starch-iodine complex change with the nature and composition of the starch, namely the amylose/amylopectin ratio [12]. Here we adopted the same detection wavelength of the original method (580 nm) [5], which is associated with an amylopectin/amylose ratio of 80:20 [12]. This can be regarded as a standard approach since this is a common composition ratio found in different starches [13]. In the case of using a particular starch as a substrate, this factor should be studied in detail and the method should be adjusted accordingly.

Another import aspect was to maximize the information about enzyme role in the hydrolysis process, such as the monitoring of the release of glucose monomers from pure starches that can be obtained when the hydrolysis is catalyzed by amyloglucosidase. To this end, we replicated the protocol used for the determination of enzyme activity, replacing the soluble starch by a pure starch (starches from different botanical sources are commercially-available). When compared with the use of soluble starch, we adjusted the volume of HCl for stopping the reaction (200 μL instead of 750 μL) in order to maintain the buffering capacity of the GOPOD reagent (glucose yield protocol, step 4). In our particular case [8], this study complemented our previous assessment of enzyme activity by the starch-iodine method.

#### Analytical performance and application

The characterization of both assays for the evaluation of the activity of amylolytic enzymes and glucose yield considered its linear range, sensitivity, repeatability, and limit of detection (LOD).

**Fig. 5:**
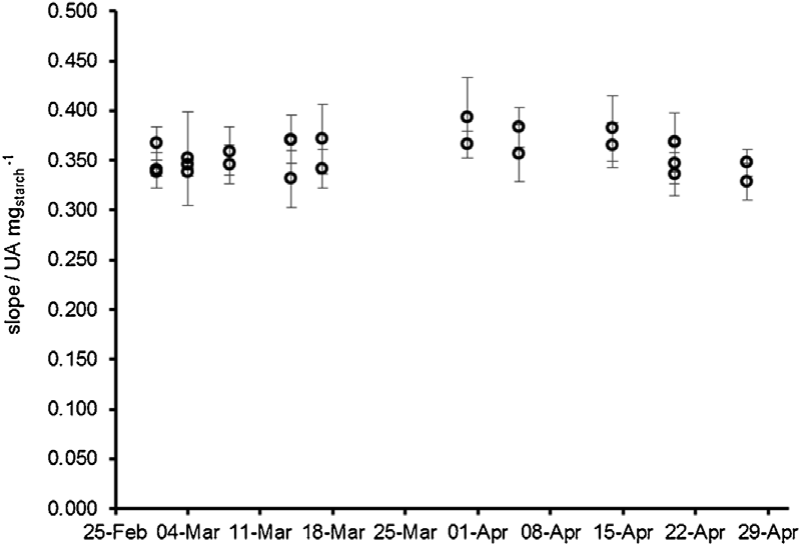
Evolution of sensitivity (represented by the slope of the calibration curve) along time for the starch-iodine assay.

**Fig. 6:**
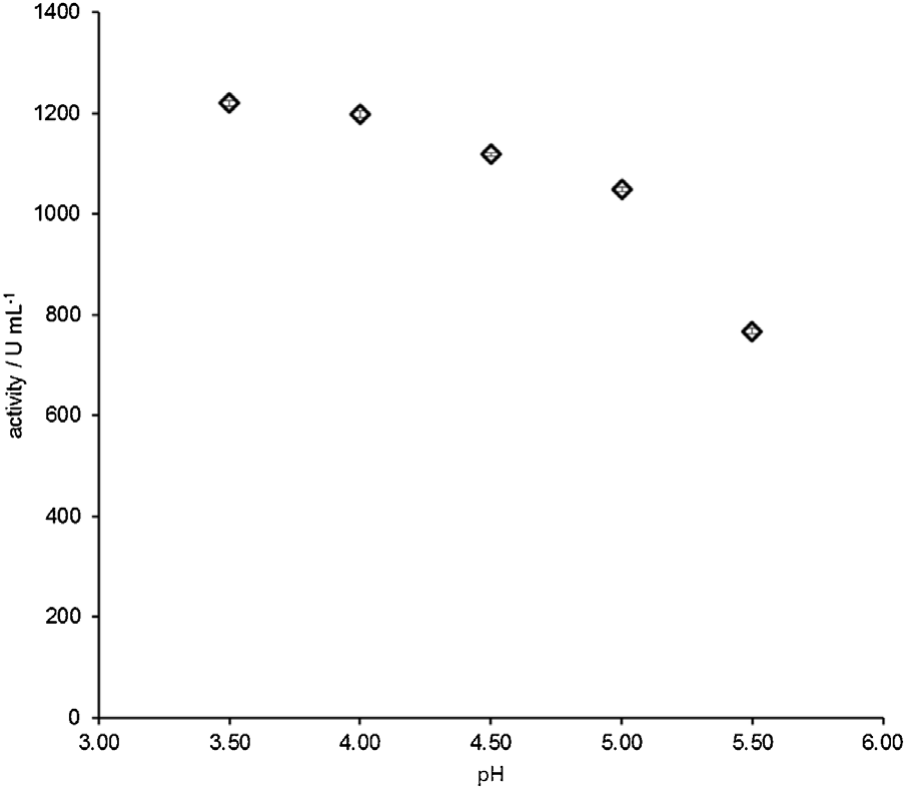
Influence of pH in the activity of an amyloglucosidase from *Aspergillus niger* (PN: A9913, Sigma Aldrich) measured by the described starch-iodine assay. Experimental conditions as described in the protocol (the incubation temperature was 45 °C).

Regarding the starch-iodine method, it was possible to measure masses of starch up to 3.00 mg (this mass respects to the total amount of starch contained in the vial, corresponding to a maximum mass of 42.9 μg in an individual microplate well). An illustrative linear (R^2^ = 0.999) calibration curve is abs_580nm_ = 0.061 (±0.025) + 0.366 (±0.014) m_starch_. The values in parentheses are the limits of the 95% confidence levels intervals, and starch mass is expressed in mg. Relative standard deviation (expressed in percentage, RSD%) measured the precision of the assay. Values were below 5% in all cases (in most of the determinations the values were below 3%). We also analyzed the reproducibility of the method by compiling the evolution of its sensitivity (defined by the slope of the calibration curve) at both intra- and inter-day in 10 experimental days during a period of approximately two months (Fig. 5). The intra-day variation of sensitivity ranged between 2.0 and 6.3%, and the inter-day variation was 4.9% for the complete period. LOD was calculated as the starch mass equivalent to the absorbance that resulted from the addition of the blank signal (standard solution without soluble starch) to 3 times the corresponding standard deviation [14]. For the proposed method, the minimum detectable amount of starch (LOD) was 0.4 mg. An example of the application of this assay is illustrated by Fig. 6, where we studied the influence of pH on the activity of amyloglucosidase from *A. niger*.

**Fig. 7:**
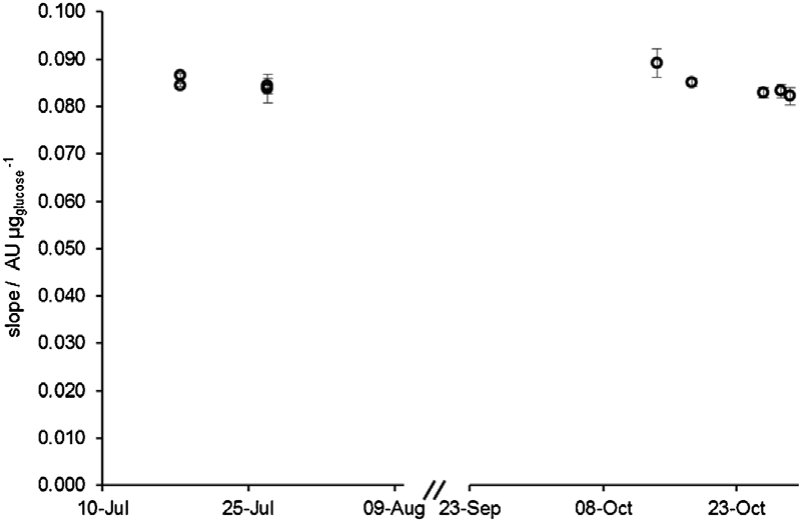
Evolution of sensitivity (represented by the slope of the calibration curve) along time for the glucose oxidase/peroxidase assay.

**Fig. 8:**
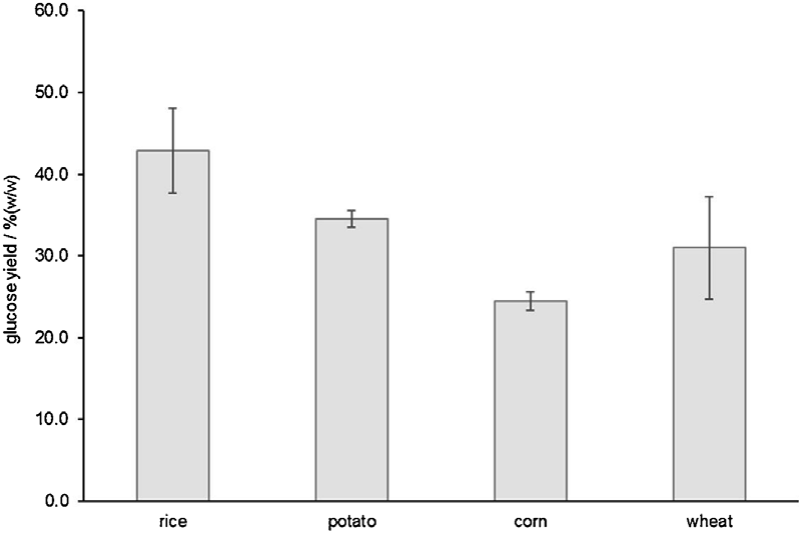
Glucose yield (average values from two determinations) measured by the described glucose oxidase/peroxidase assay that resulted from the hydrolysis of four different pure starches mediated by an amyloglucosidase from *Aspergillus niger* (PN: A9913, Sigma Aldrich).

For the GOPOD method under microplate format, we also performed a similar method characterization. It was possible to linearly (R^2^ ≥ 0.999) measure masses of glucose up to 10.0 μg (this corresponds to the actual mass present in the microplate well, corresponding to 50 μL of a 200.0 mg L^−1^ of a glucose standard solution). In these experimental conditions, an illustrative calibration curve is abs_505nm_ = 0.042 (±0.005) + 0.0846 (±0.0008) m_glucose_. For this assay, the values in parentheses also correspond to the limits of the 95% confidence levels intervals, and glucose mass is expressed in μg. This assay was also very precise, with RSD values below 3% in all cases. We also monitored the sensitivity of this method along 7 different days in 2 different months (Fig. 7). The intra-day variation was below 1.6%. The RSD value for inter-day precision was 2.5%. Finally, by using the same criteria applied to the previous method, the calculated LOD for this assay was 0.06 μg of glucose. An example of the application of this method was the determination of the glucose yield after the hydrolysis of four pure starches assisted by an amyloglucosidase from *A. niger* (Fig. 8) [8].

## Conclusion

The present method describes an improved experimental protocol that can be used to monitor the activity of two amylolytic enzymes: alpha-amylase and amyloglucosidase. This method intends to facilitate the implementation of a new routine determination of alpha-amylase and amyloglucosidase activities in any laboratory that could deal with amylolytic enzymes by providing all the necessary experimental details and conditions that could maximize the performance of the method. The long-term use of the method also showed a high-precision for both intra-day and inter-day measurements. In the case of amyloglucosidase, it is also possible to measure glucose yield using a protocol similar to the one applied to the enzyme activity. Furthermore, this improved method is very flexible and allows a broadband application, being able to be easily adjusted according to the requirements of each study or the equipment and instrumentation available at each laboratory.

## Appendix A Extraction of amylolytic enzymes

Another potential application of the proposed method is the evaluation of the activity of amylolytic enzymes extracted from different natural matrices such as flours and microbial enzyme preparations. These extractions are commonly performed with buffer or salt solutions at mild temperatures (from room temperature to 40 °C). As an example, we summarize in this section the extraction protocol for the measurement of alpha-amylase activity in wheat flour [9]. More details and information about the extraction of other amylolytic enzymes from different natural matrices can be found in the method AOAC 2002.01, commonly named in the literature as the Ceralpha method [9].

### Preparation of the extraction buffer (concentrated sodium malate buffer−1 M, pH 5.4)

Dissolve 134.1 g of malic acid, 70 g of NaOH, and 58.4 g of NaCl in 900 mL of water (deionized or ultra-pure). Next, add 6.0 g CaCl_2_⋅2H_2_O until complete dissolution. The pH should be adjusted to pH 5.4 by dropwise addition of concentrated (4 mol L^−1^) NaOH or HCl. Sodium azide (1.0 g) can be added as a preservative (antimicrobial agent). If sodium azide is added, the reagent will be stable for more than one year. If not, the shelf-life of the reagent will be 2 weeks if preserved at 4 °C. This concentrated buffer should be diluted 200 times (*e.g.* 50 mL of concentrated solution for a final volume of 1000 mL) in order to be used in the extraction protocol.

### Extraction of alpha-amylase from wheat flour

After weighing 3.0 g of flour into a 50 mL flask, add 20.0 mL malate buffer pH 5.4 (diluted from the concentrated solution). The flask should be vigorously swirled and placed in a temperature-controlled water bath for 15–20 min at 40 °C with occasional swirling. Proceed as described in steps 4–8 of the amylolytoc enzyme activity assay protocol. The enzyme activity should be determined within one hour.

## Appendix B Illustrative calculations

This section illustrates an example of the application of the method that includes the corresponding calculations for both methods herein described.

### Amylolytic enzyme activity assay

For the enzyme amyloglucosidase from *Aspergillus niger* (PN: A9913, Sigma Aldrich), 10 μL of the commercial enzyme solution were mixed with 1990 μL of universal buffer at the desired pH (enzyme solution 1, dilution factor 200). This was followed for a second dilution of 500 μL of the previous solution with 4500 μL of universal buffer at the desired pH (enzyme solution 2, dilution factor 10). Hence, the dilution factor corresponded to 2000. Next, for the assay experiment, 500 μL of enzyme solution 2 was mixed in a vial that contained 1500 μL of a soluble starch solution 2.00 g L^−1^ and 1000 μL of universal buffer. For the control experiment, the vial contained 1500 μL of soluble starch solution 2.00 g L^−1^ and 1500 μL of universal buffer. Both recipients were placed in a heating block for 10 min at a temperature of 45 °C. After 10 min, the hydrolysis was stopped with the addition of 750 μL of 1 mol L^−1^ HCl. Then, 50 μL of each vial were transferred to the 96-well microplate, followed by the addition of 150 μL of water and 50 μL KI/I_2_ reagent. The absorbance at 580 nm was measured for both assay and control experiments for 10 min, being the average values of 0.110 and 1.138, respectively. By intercepting those absorbance values in the calibration curve (*e.g.*, abs_580nm_ = 0.061 (±0.025) + 0.366 (±0.014) m_starch_), we found that the mass of starch after hydrolysis (m_assay_) was 0.133 mg, and the mass of starch of the control (m_control_) experiment was 2.94 mg, respectively. Regarding that the enzyme is absent for the control experiment, it is expectable that the absorbance of the control experiment would be similar to the most concentrated standard of soluble starch prepared (3.00 mg, Table 1). Therefore, our variables for Eq. (1) are: m_control_ = 2.94 mg, m_assay_ = 0.133 mg, time = 10 min, volume of enzyme used in the assay = 0.500 mL, dilution factor = 2000. By applying Eq. (1) the calculated activity for this enzyme was 1123 U mL^−1^.

### Glucose yield assay

For the calculation of the glucose yield assay promoted by amyloglucosidase from *Aspergillus niger* (PN: A9913, Sigma Aldrich), 1500 μL of water were added to a vial containing of ˜3.00 mg of a pure rice starch (m_starch_) weighed in an analytical scale. For this particular case, m_starch_ was 2.40 mg. Next, 500 μL of enzyme solution 2 (described in detail in the previous example) and 1000 μL of universal buffer were added to the vial (in this particular case, the objective was to obtain extra information about the hydrolysis performance of the enzyme and for this reason we used the same enzyme dilution and mass of starch used for the enzyme activity method. These parameters should be adjusted according to the objectives of each individual study). The vial was then placed for 10 min into a heating block at 45 °C. After 10 min, the reaction was stopped with 200 μL of HCl 1.0 mol L^−1^ and the vial was centrifuged for 5 min at 350 g. Next, 50 μL of supernatant were transferred to a 96-well microplate, followed by the addition of 150 μL of GOPOD reagent. The absorbance of the well was monitored at 505 nm, and the value recorded was 0.681. By intercepting this absorbance values in the calibration curve (*e.g.* abs_505nm_ = 0.042 (±0.005) + 0.0846 (±0.0008) m_glucose_), we obtained a mass of glucose of 7.56 μg that is contained in the 50 μL of sample transferred to the microplate. This corresponds to a total mass of 483.4 μg of glucose (m_glucose_) in the 3200 μL total volume. Therefore, the glucose yield corresponded to the ratio between m_glucose_ (483.4 μg) and the total mass of starch m_starch_ (2400 μg), which resulted in a value of 20.14%. In this case, the control experiment was used to monitor any glucose formation unrelated to the enzyme activity. Therefore, the control assay should provide glucose yields of ˜0%.

## References

[1] Europe S. (2018). Starch Europe – Key Figures 2016. https://www.starch.eu/the-european-starch-industry/#figures.

[2] Gangadharan D., Sivaramakrishnan S., Singh-Nee Nigam P., Pandey A. (2009). Amylolytic enzymes. Biotechnology for Agro-Industrial Residues Utilisation: Utilisation of Agro-Residues.

[3] Miller G.L. (1959). Use of dinitrosalicylic acid reagent for determination of reducing sugar. Anal. Chem..

[4] Fuwa H. (1954). A new method for microdetermination of amylase activity by the use of amylose as substrate. J. Biochem..

[5] Xiao Z.Z., Storms R., Tsang A. (2006). A quantitative starch-iodine method for measuring alpha-amylase and glucoamylase activities. Anal. Biochem..

[6] Somogyi M. (1952). Notes on sugar determination. J. Biol. Chem..

[7] Oliveira H.M., Correia V.S., Segundo M.A., Fonseca A.J.M., Cabrita A.R.J. (2017). Does ultrasound improve the activity of alpha amylase? A comparative study towards a tailor-made enzymatic hydrolysis of starch. LWT.

[8] Oliveira H.M., Pinheiro A.Q., Fonseca A.J.M., Cabrita A.R.J., Maia M.R.G. (2018). The intensification of amyloglucosidase-based saccharification by ultrasound. Ultrason. Sonochem..

[9] McCleary B.V., McNally M., Monaghan D., Mugford D.C. (2002). Measurement of alpha-amylase activity in white wheat flour, milled malt, and microbial enzyme preparations, using the Ceralpha assay: collaborative study. J. AOAC Int..

[10] Xiao Z., Storms R., Tsang A. (2007). Corrigendum to “A quantitative starch–iodine method for measuring alpha-amylase and glucoamylase activities” [Anal. Biochem. 351 (2006) 146–148]. Anal. Biochem..

[11] Mäntele W., Deniz E. (2017). UV–vis absorption spectroscopy: Lambert-Beer reloaded. Spectrochim. Acta Part A-Mol. Biomol. Spectrosc..

[12] Jarvis C.E., Walker J.R.L. (1993). Simultaneous, rapid, spectrophotometric determination of total starch, amylose and amylopectin. J. Sci. Food Agric..

[13] Nalin T., Sperb-Ludwig F., Venema K., Derks T.G.J., Schwartz I.V.D. (2015). Determination of amylose/amylopectin ratio of starches. J. Inherit. Metab. Dis..

[14] Miller J.N., Miller J.C. (2010). Statistics and Chemometrics for Analytical Chemistry.

